# Laboratory and field evaluation of acetic acid-based lures for male Asian citrus psyllid, *Diaphorina citri*

**DOI:** 10.1038/s41598-019-49469-3

**Published:** 2019-09-09

**Authors:** Odimar Z. Zanardi, Haroldo X. L. Volpe, Rejane A. G. Luvizotto, Rodrigo F. Magnani, Francisco Gonzalez, Carolina Calvo, Cameron A. Oehlschlager, Benjamin J. Lehan, Victoria Esperança, Jennifer Y. Delfino, Renato de Freitas, Rômulo Igor de Carvalho, Tatiana Aparecida Mulinari, Marcelo P. Miranda, José Mauricio S. Bento, Walter S. Leal

**Affiliations:** 1 Research and Development Department, Fund for Citrus Protection (Fundecitrus), Vila Melhado, Araraquara, SP 14807-040 Brazil; 2 Chemtica International S.A, Santo Domingo, Heredia, Apdo 640-3100 Costa Rica; 3 California State Polytechnic University-Pomona, 3801W. Temple Avenue, Pomona, CA 91768 USA; 4 Department of Entomology and Acarology, Luiz de Queiroz College of Agriculture, University of São Paulo (ESALQ/USP), Piracicaba, SP 13418-900 Brazil; 5 Department of Molecular and Cellular Biology, University of California-Davis, Davis, CA 95616 USA

**Keywords:** Behavioural ecology, Entomology

## Abstract

The Asian citrus psyllid (ACP) is a vector of a pathogen associated with greening and thus a major problem in citriculture worldwide. Lures are much needed for improving ACP trapping systems for monitoring populations and surveillance. Previously, we have identified acetic acid as a putative sex pheromone and measured formic acid- and propionic acid-elicited robust electroantennographic responses. We have now thoroughly examined in indoor behavioral assays (4-way olfactometer) and field tests the feasibility of these three semiochemicals as potential lures for trapping ACP. Formic acid, acetic acid, and propionic acid at appropriate doses are male-specific attractants and suitable lures for ACP traps, but they do not act synergistically. An acetic acid-based homemade lure, prepared by impregnating the attractant in a polymer, was active for a day. A newly developed slow-release formulation had equal performance but lasted longer, thus leading to an important improvement in ACP trap capture at low population densities.

## Introduction

The Asian citrus psyllid (ACP), *Diaphorina citri* Kuwayama (Hemiptera: Liviidae) is the most devastating problem to the citrus industry worldwide^1^. ACP is the major vector of the ‘*Candidatus* Liberibacter spp.’, which is transmitted from tree to tree and causes the citrus greening, also known as Huanglongbing (HLB). HLB has caused a severe impact on the citrus industry in China, the United States, and Brazil^2^. In Brazil, about one-fourth of the citrus trees in the State of São Paulo has been eradicated since 2004 as part of an HLB control strategy^3^. In the United States, Florida citriculture has sustained severe losses, whereas in Arizona and California surveillance is sorely needed because the vector (ACP) is present while HLB has been detected, but not established in commercial orchards^2^. Of note, 1,100 findings of HLB in urban, but not in commercial orchards^4^, suggest that the disease is already established in urban areas in California. Monitoring ACP populations is essential for integrated vector management and, more importantly, for surveillance. One of the challenges for abatement personnel in areas of low ACP densities is to capture the vector to determine infection status so that control strategies can be implemented before HLB is spread. Therefore, the development of trapping systems is at a premium, particularly the discovery of lures for enhancing ACP captures in areas of low populations. Previously, we have identified acetic acid as a putative ACP sex pheromone, in addition to other related compounds that also generated electroantennographic (EAG) responses^5,6^. Here, we report thorough behavioral and field tests that led us to conclude that formic acid and propionic acid are also male attractants. Additionally, we show that an acetic acid-based, slow-release formulation can be implemented for monitoring ACP populations.

## Results and Discussion

### Measuring attraction elicited by EAG-active compounds

**Figure 1:**
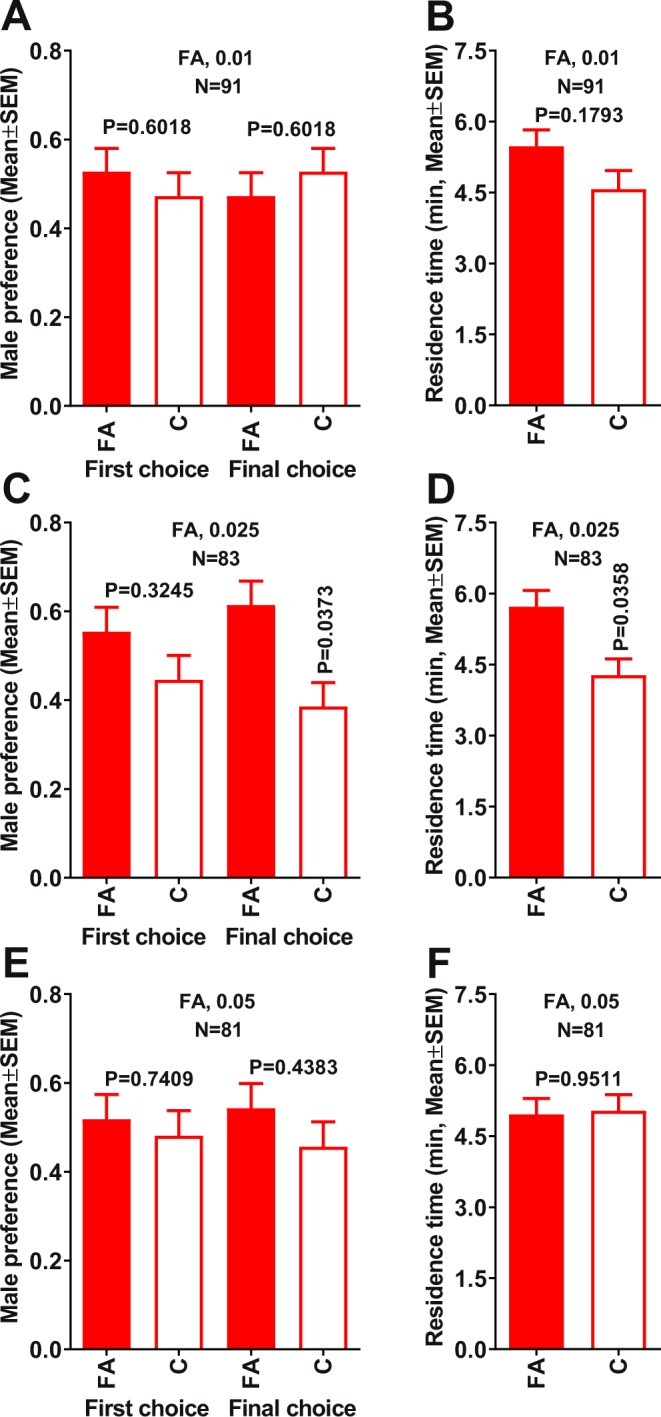
Behavioral responses of 7-day-old virgin males to formic acid in a 4-way olfactometer. Male first and final choices when comparing (**A**) 0.01, (**C**) 0.025, and (**E**) 0.05 µg of formic acid with control. For consistency, all related figures are presented in increasing doses from top to bottom. Comparison of residence times in each treatment vs. control with formic acid at (**B**) 0.01, (**D**) 0.025, and (**F**) 0.05 µg. Male preference indicates the proportion of males making first and final choice. Means were compared by Wilcoxon matched-pairs signed-rank tests. FA = formic acid.

Using a 4-way olfactometer, we tested whether ACP males and/or females were attracted to compounds previously shown to elicit robust EAG responses from male and female antennae, namely, formic and propionic acids^6^. Because these semiochemicals were not detected in earlier headspace collections from separated groups of ACP males and females^6^, we surmised that they could be released at doses lower than acetic acid, which was previously demonstrated to be an attractant when tested at 1 µg (source dose)^6^. In preliminary olfactometric tests with formic acid at 0.5 and 0.1 µg doses no significant differences were observed in male responses to treatment and control (*n* = 80, first choice, P = 0.1456; final choice, P > 0.9999; residence time, P = 0.5194 and *n* = 82, first choice, P = 0.9192; final choice, P > 0.9999; residence time, P = 0.6594, respectively). Likewise, no significant differences were observed when formic acid was tested at 0.05 µg (Fig. 1E,F). At a lower dose (0.025 µg), however, in their final choice (but not in the first choice) males showed a significant preference for the sidearm of the arena with formic acid as compared to the control side (Fig. 1C). They spent significantly longer time in the treatment sidearm of the arena (Fig. 1D). Attractivity was lost by further decreasing the dose (to 0.01 µg) (Fig. 1A,B). ACP females showed no significant preference for formic acid in any of the tested doses (Fig. S1). We, therefore, concluded that formic acid at appropriate doses is an ACP male attractant.

**Figure 2:**
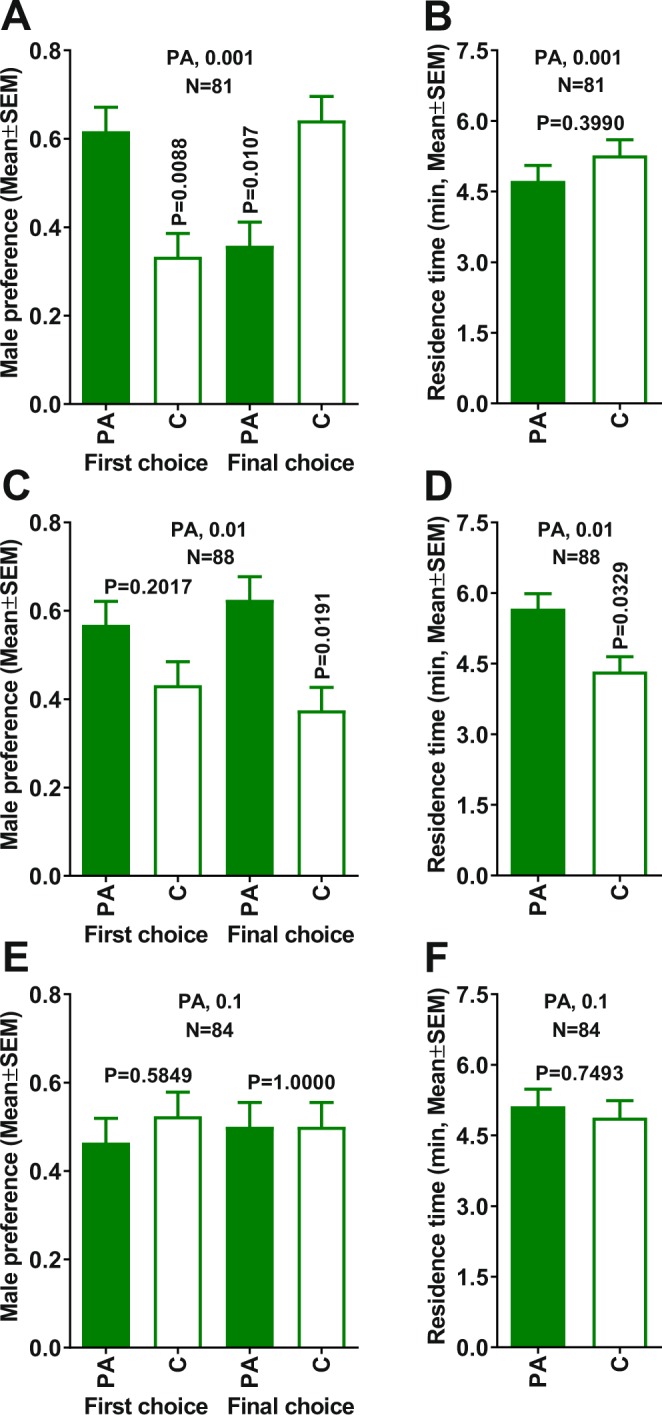
Behavioral responses of 7-day-old virgin males to propionic acid in a 4-way olfactometer. Male first and final choices when comparing treatments with (**A**) 0.001, (**C**) 0.01, and (**E**) 0.1 µg of propionic acid vs. control. Comparison of residence times in each treatment vs. control with propionic acid at (**B**) 0.001, (**D**) 0.01, and (**F**) 0.1 µg. Male preference indicates the proportion of males making first and final choice. Means were compared by Wilcoxon matched-pairs signed-rank tests. PA = propionic acid.

Preliminary olfactometric experiments with propionic acid at 0.5 µg dose, showed no significant male attraction (*n* = 75, first choice, P > 0.9999; final choice, P > 0.9999; residence time, P = 0.7821). Likewise, no male attraction was observed when propionic acid was tested at 0.1 µg (Fig. 2E,F). With a 10x lower dose, ie, 0.01 µg, ACP males showed a significant preference for the sidearm of the arena treated with propionic acid compared to control as indicated by the final, but not the first choice (Fig. 2C). Additionally, males spent significantly more time in the treatment than in the control side of the arena (Fig. 2D). At a lower dose (0.001 µg), ACP males showed a significant preference for the treatment side of the arena in the first choice (Fig. 2A); however, in their final choice, males showed a significant preference for the control side of the arena (Fig. 2A). In addition, the residence time in the control and propionic acid arms were similar at 0.001 µg (Fig. 2B). By contrast, ACP females showed no attraction whatsoever to propionic acid at the tested doses (Fig. S2). In conclusion, olfactometer experiments suggested that propionic acid is also a male attractant.

**Figure 3:**
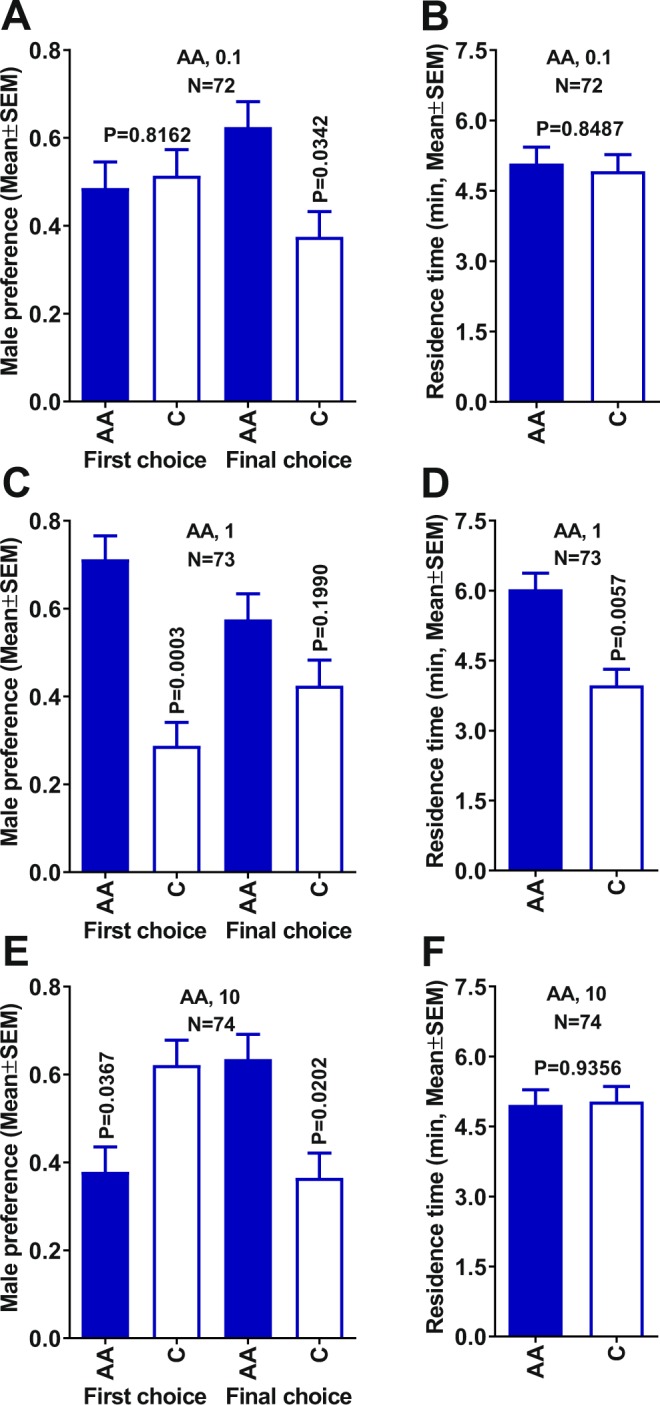
Behavioral responses of 7-day-old virgin males to acetic acid in a 4-way olfactometer. Male first and final choices when comparing treatments with (**A**) 0.1, (**C**) 1, and (**E**) 10 µg of acetic acid vs. control. Comparison of residence times in each treatment vs. control with acetic acid at (**B**) 0.1, (**D**) 1, and (**F**) 10 µg. Male preference indicates the proportion of males making first and final choice. Means were compared by Wilcoxon matched-pairs signed-rank tests. AA = acetic acid.

Next, we tested whether the narrow range of activity observed with formic and propionic acids in olfactometer measurements was also manifested with acetic acid. Previously, we tested acetic acid at 1 µg so this time we compared this dose with lower and higher decadic doses. At 0.1 µg dose, there was no significant differences manifested in the first choice (Fig. 3A) or residence time (Fig. 3B). However, ACP males showed some preference for the treatment as indicated by the final choice (Fig. 3A). Behavioral measurements at 1 µg of acetic acid corroborate previous findings^6^ indicating that males had a significant preference for the treatment side of the arena in their first choice (Fig. 3C) and residence time (Fig. 3D). It is noteworthy that this time, we measured the final choice and found no significant difference between treatment and control (Fig. 3C). At a higher dose (10 µg), ACP males showed a significant preference for the treatment as indicated in their final, but not in the first choice (Fig. 3E), or the residence time (Fig. 3F). Males showed a significant preference for the control in their first choice, thus suggesting a possible repellent (deterrent) effect at high concentration. These findings underscore the sensitive nature of dose-dependence and, therefore, the importance of developing formulations that release a precise dose of this attractant under field conditions. Measurement of female response under the same conditions showed that females are not attracted to acetic acid at the tested doses (Fig. S3). These findings are consistent with previous observations using both Y- and 4-way olfactometers that led us to conclude that acetic acid is not a female attractant^6^.

### Field evaluations in Brazil of attractants as potential ACP lures

**Figure 4:**
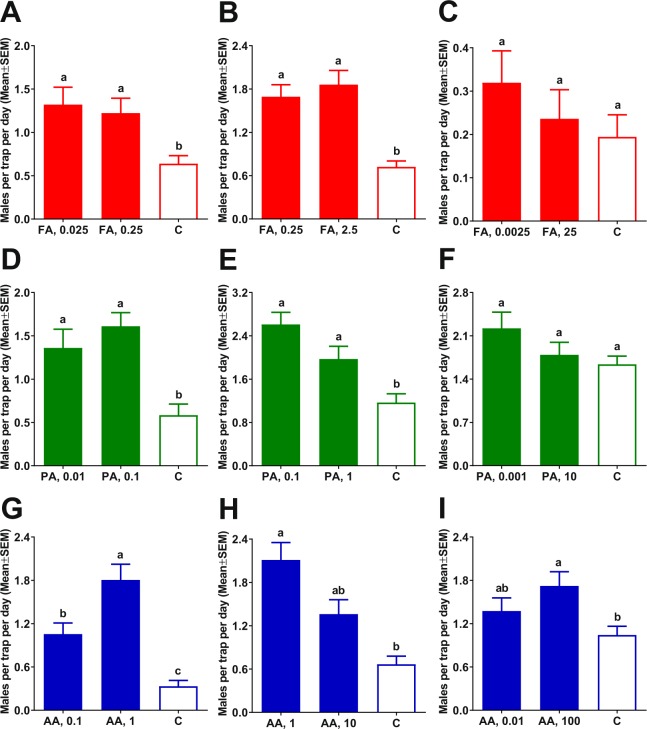
Field evaluations of ACP attractants as potential lures. Male captures in traps baited with formic acid (FA) at (**A**) 0.025 and 0.25 µg; (**B**) 0.25 and 2.5 µg; (**C**) 0.0025 and 25 µg; propionic acid (PA) at (**D**) 0.01 and 0.1 µg; (**E**) 0.1 and 1 µg; and (**F**) 0.001 and 10 µg; and acetic acid (AA) at (**G**) 0.1 and 1 µg; (**H**) 1 and 10 µg; and (**I**) 0.01 and 100 µg were compared with their respective controls. Data were analyzed by Kruskal-Wallis, followed by Dunn’s multiple comparison tests. In each test, bars labeled with the same letters are not significantly different.

Behavioral measurements in olfactometer suggested that, in addition to acetic acid, formic and propionic acids too are ACP male attractants. To determine their potential use as lures for trapping ACP males, we tested under field conditions each one of these compounds in their optimal doses (as determined by the above indoor assays), and compared them with lower and higher doses. To minimize the possibility that physical stimuli from trapped males may lead to the capture of females, we changed the protocol for these field experiments, ie, all captured males were removed daily (as opposed to weekly, as previously^6^ done). Additionally, we limited each treatment to a 3 × 3 Latin square, with one control and two treatments. First, we compared formic acid at its optimal dose (0.025 µg) for an olfactometer assay with a 10x higher dose (0.25 µg) (Fig. 4A). Traps baited with formic acid at both doses captured significantly more ACP males than control traps, and there was no significant difference between these two treatments (Fig. 4A). Next, we compared 0.25 µg with a 10x higher dose (2.5 µg) (Fig. 4B). Traps baited with formic acid captured significantly more ACP males than control traps, and there was no significant difference between doses. Lastly, we tested traps baited the lowest (0.0025 µg) and the highest doses (25 µg), but there were no significant differences between catches in either of these traps as compared to captures in control traps (Fig. 4C). In summary, trap baited with formic acid at doses from 0.025 to 2.5 µg captured significantly more ACP males (Fig. 4A,B).

Using the same evaluation protocol, we next compared ACP captures in traps baited with 0.1 and 0.01 µg of propionic acid. Traps loaded with these doses of propionic acid captured significantly more ACP males than control traps (Fig. 4D). We then compared 0.1 µg with a 10x higher dose (1 µg). Again, traps baited with propionic acid captured significantly more ACP males than control traps, and there was no significant difference between doses (Fig. 4E). Lastly, we compared the performance of traps baited with the lowest (0.001 µg) and the highest (10 µg) doses with control traps. Captures in these traps were not significantly different from catches in control traps (Fig. 4F), thus suggesting that propionic acid is an effective lure at doses ranging from 0.01 to 1 µg.

Previous preliminary experiments showed that traps baited with 1, 10, or 100 µg of acetic acid captured more ACP males than control traps^6^. We have now tested various doses of acetic acid using the same above-described protocol that restricts the experimental design to 3 × 3 Latin square and calls for the removal of trapped insects daily. Surprisingly, acetic acid at 0.1 µg captured significantly more ACP males than control traps (Fig. 4G). Consistent with our previous findings^6^, traps baited with acetic acid at 1 µg captured significantly more ACP males than traps loaded with 0.1 µg of acetic acid as well as control traps (Fig. 4G). Captures in traps loaded with a higher dose of acetic acid (10 µg) did not perform significantly better than traps baited with 1 µg (Fig. 4H). Likewise, traps with a very low dose (0.01 µg) did not differ significantly from control traps (Fig. 4I). Traps with 100 µg of acetic acid performed slightly, but significantly, better than control traps (Fig. 4I). In conclusion, acetic acid is an ACP male attractant, but it is effective at a narrow range of doses.

None of the traps baited with formic, acetic, or propionic acids (same doses tested for males) caught significantly more ACP females than their respective control traps (Fig. S4). As previously discussed^6^, earlier observations of higher captures of females in acetic acid-baited traps than in control traps might be an artifact possibly derived from physical stimuli from captured males. By removing trapped males daily (as opposed to weekly collections), we were able to unambiguously conclude that acetic acid is not a female attractant. Likewise, formic and propionic acids are male-specific attractants.

### Field evaluation in Brazil of lures for possible synergy

**Figure 5:**
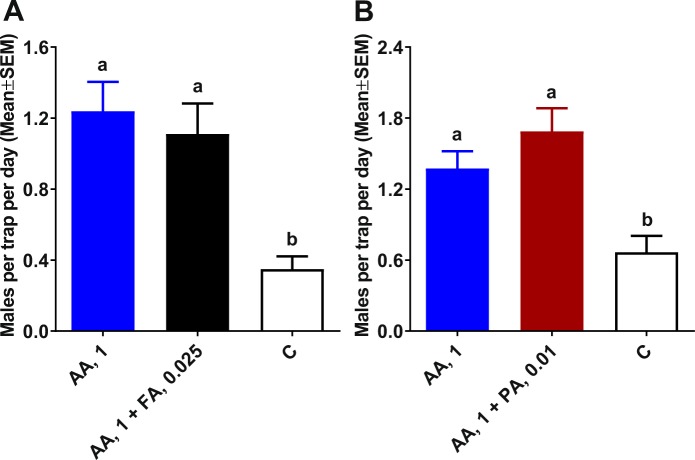
Field evaluations of potential synergy between attractants. Captures in traps baited with acetic acid (AA) 1 µg were compared with catches of ACP males in traps baited with a (**A**) binary mixture of AA, 1 µg plus formic acid (FA), 0.025 µg or (**B**) a binary mixture of AA, 1 µg plus propionic acid (PA), 0.01 µg and their respective control traps. Data were analyzed by Kruskal-Wallis, followed by Dunn’s multiple comparison tests. For each test, bars labeled with the same letters are not significantly different.

To explore possible synergy between lures, traps baited with 1 µg of acetic acid were compared with those loaded with a mixture of either acetic acid plus formic acid or acetic acid plus propionic acid. No synergy was observed when 1 µg of acetic acid was compared to 1 µg of acetic acid plus formic acid at its optimal dose (0.025 µg) (Fig. 5A). Traps baited with this binary mixture or loaded with acetic acid alone captured significantly more ACP males than control traps. However, there was no significant difference between acetic acid alone and acetic acid in combination with formic acid (Fig. 5A). Attempts with higher doses of formic acid, which were effective when used alone (Fig. 4A–C), were unrewarding (Fig. S5). Catches of ACP males in traps baited with 0.25, 0.75, 1.5, or 2.25 µg of formic acid in addition to 1 µg of acetic acid did not significantly differ from captures in traps baited with 1 µg of acetic acid alone (Fig. S5). Next, we compared captures in traps loaded with 1 µg of acetic acid alone or in combination with 0.01 µg of propionic acid. Traps baited with acetic acid alone or this binary mixture captured more ACP males than control traps, but there was no significant difference between the two types of lures (Fig. 5B). It is conceivable that these three semiochemicals act on the same receptor, which might not be so narrowly tuned as to discriminate the same functional group (acid) with 1, 2, or 3 carbons.

### Field tests of slow-release formulations in California

**Figure 6:**
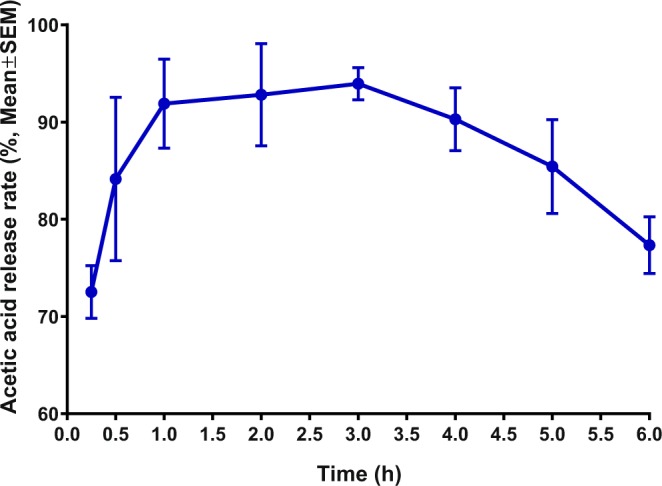
Relative amounts of acetic acid released from a homemade lure. One hundred microliters of a solution of acetic acid in hexane (0.01 mg/ml) were loaded on an ethylene-propylene side-by-side device. After solvent evaporation (5 min), headspace volatiles were captured for 1 min with HiSorb and analyzed by TD-GC/MS with single-ion monitor (m/z 61). *n* = 3.

Preliminary tests conducted at the campus of CalPoly-Pomona on July 2018 showed that traps loaded with the newly developed lure (ChemTica-A) captured significantly more ACP males than control traps (n = 24, control, 0.5 ± 0.1; treatment, 1.87 ± 0.34 males per trap per day; P = 0.0001, Mann-Whitney test). Next, we aimed at comparing ChemTica-A with a standard lure. Typically, the performance of newly developed lures is compared with natural sources (eg, caged virgin females vs. newly synthesized sex pheromone system). This is not feasible in ACP case, because placing caged females in the field as lures did not lead to a significant increase in captures as compared to control traps despite the observation that males are attracted to virgin females in Y- and 4-way olfactometers^6^. Therefore, we used our standard homemade formulation (comprised of a disk made of ethylene-propylene side-by-side impregnated with acetic acid) for comparison with ChemTica-A. First, we determined the longevity of our homemade lure. Using HiSorb™, we collected headspace from freshly-prepared homemade lures at different intervals for 6 h, and analyzed the samples by Thermal Desorption, Gas Chromatography linked to a Mass Spectrometer (TD-GC/MS). These analyses showed that our homemade lures reached a peak performance between 1 and 4 hours after preparation, with a clear decay after 6 hours (Fig. 6).

**Figure 7:**
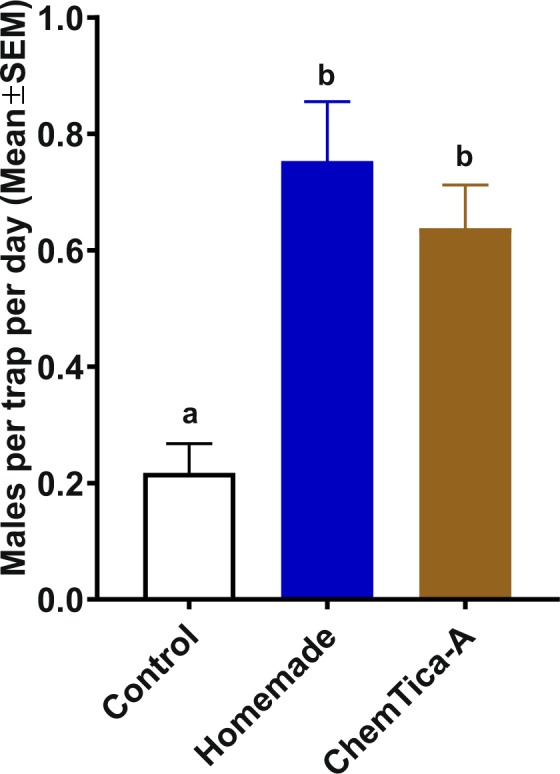
Comparison of a newly developed slow-release formulation (ChemTica-A) with a homemade formulation that required daily renewal. Despite ACP low density in the tested area, traps baited with slow-release and homemade formulations captured significantly more ACP males than control traps. Data were analyzed by Kruskal-Wallis, followed by Dunn’s multiple comparison tests. Bars labeled with the same letter represent treatments that are not significantly different.

In these field experiments, all traps were inspected daily, insects were removed, and freshly prepared homemade lures were attached to traps at the beginning of ACP flight activity (10–11 AM), whereas ChemTica-A lures were used for the duration of these tests. Results from field tests at the CalPoly campus in September 2018, demonstrated that both ChemTica-A and homemade lures captured significantly more ACP males than control traps (Fig. 7). There was no significant difference between the captures with homemade lures renewed daily, and ChemTica-A lures used throughout these tests (Fig. 7). Of note, ACP density at the time of these experiments was very low so that it was almost impossible for the experimenters to determine the presence of the insect by visual inspection of orange trees. ACP visual inspections were made daily in the same plants where yellow traps were installed, by observation of the presence/absence of nymphs and landed adults in five flushes per side of the tree canopy.

In summary, formic, acetic, and propionic acid are practical ACP lures. Of these, acetic acid has the widest, albeit narrow, range of active doses, thus facilitating the development of slow-release formulations. Here, we show that a newly developed formulation (ChemTica-A) significantly improved yellow sticky trap performance. When tested in an area of low ACP density, traps baited with ChemTica-A capture in average 3x more ACP males than naked yellow sticky traps. Therefore, ChemTica-A formulation has potential applications in surveillance programs aimed at earlier detection of infectious psyllids. Further research is needed to determine whether captures in ChemTica-A-baited traps faithfully correlate with psyllid population densities and thus serve as a tool to guide control measures.

## Materials and Methods

### Insect preparations

A laboratory ‘*Ca*. Liberibacter spp.’ free ACP colony was initiated with insects collected from *Murraya paniculata* (L.) Jack (Sapindales: Rutaceae) in São Paulo State, Brazil in 2009 and reared in a greenhouse under 25 ± 5 °C. For this purpose, 400 mated *D*. *citri* adults were placed in cages (60 × 60 × 60 cm) containing *M*. *paniculata* seedlings with new flushes and kept for seven days for oviposition. Then, adults were removed and the seedlings with eggs were kept in cages. Once nymphs reached the fifth instar, cages were transferred to a climate-controlled room at 25 ± 2 °C, 65 ± 10% RH, L14: D10 h photoperiod, and 3000 lux luminosity until adult emergence. Newly emerged adults were collected and separated by gender for olfactometric assays.

### The longevity of homemade device

The ES fibers were loaded with 100 μL of an acetic acid solution 0.01 μg/μL diluted in hexane, corresponding to the absolute amount of 1 μg acetic acid. Then, ES fibers were placed inside 20 mL glass vials (Markes International Ltd., Llantrisant, UK). After five min, the vials were sealed with silicone/polytetrafluoroethylene septs and brass caps. The sorbtive extraction probes coated with polydimethylsiloxane (HiSorb^TM^, Markes International Ltd., Llantrisant, UK), used to trap acetic acid, were pre-conditioned during 1 h at 280 °C under 1 mL/min of nitrogen flow. The probes were inserted into the glass vials to collect acetic acid released by the ES fibers during 1 min. These assays were done in the same climate-controlled room conditions as those for behavioral assays. The probes used to trap acetic acid were inserted into empties inert-coated stainless-steel thermal desorption tubes (Markes International Ltd., Llantrisant, UK) and trapped acetic acid was analyzed by TD-GC-MS. Acetic acid was thermally desorbed from the probes in an ULTRA-xr thermal desorption (TD) unit with automatic sampler UNITY-xr (Markes International Ltd., Llantrisant, UK) at 200 °C for 10 min under a helium flow rate of 50 mL/min being concentrated on a cold trap (ref. U-T11GPC-2S, general-purpose carbon; Markes International Ltd., Llantrisant, UK) held at 25 °C. The cold trap was desorbed at 280 °C for 5 min and the transfer line temperature was set at 200 °C, while acetic acid was released in a splitless mode to a polyethylene glycol capillary column (Rtx^®^-Wax 10 m × 0.10 mm i.d. × 0.10 μm film; Restek Corporation, Bellefonte, PA, USA). The gas chromatography coupled to a mass spectrometer was a GCMS-QP2010 Plus (Shimadzu Corporation, Kyoto, Japan). The GC temperature program consisted of start temperature at 35 °C held for 1 min, followed by a temperature rate of 3 °C/min to 100 °C, subsequently increased at a rate of 70 °C/min to 250 °C and then held for 3 min. The detector interface and the ion source temperature were 250 and 200 °C, respectively, the carrier gas was He (50 mL/min, splitless). Mass spectra were recorded at 70 eV and all analyses were done in the selected ion monitoring (SIM) mode, screening the acetic acid molecular ion *m/z* 60. Under these conditions, the retention time of standard acetic acid was 4.56 min.

### Measuring attraction elicited by EAG-active compounds

All behavioral assays were conducted in a climate-controlled room (see above). An air-flow olfactometer, also known as “4-arm olfactometer” or “4-way olfactometer” was constructed^7^ (30.0 × 30.0 × 2.5 cm in length, width, and height, respectively) with acrylic. In summary, yellow fields were made by adding a yellow laserjet printed-paper below the bottom of the arena^6^. All connections were made with 0.635-diameter polytetrafluoroethylene (PTFE) tubes (Sigma-Aldrich, Bellefonte, PA, USA). Clean air was provided by an oil-free air compressor (Schulz MSV6, Schulz, Joinville, SC, Brazil), charcoal-filtered and humidified into milliQ water before entering the olfactometer line, which was split into four separate lines, each one with an air in the 0.1 to 1.0 L/min range (Brooks Instrument, Hatfield, PA, USA) so as to allow balanced flow of 0.1 mL/min. Two of the four possible fields received volatiles from formic acid (0.01, 0.025, or 0.05 µg), acetic acid (0.1, 1, or 10 µg), or propionic acid (0.001, 0.01, or 0.1 µg), whereas the other two fields remaining received only hexane. Each one of these compounds was loaded on a cotton swab (100 µL in hexane) to give the specified total amount. New cotton swabs with loaded compounds in hexane were inserted into the headspace chambers for each tested insect. Seven-day-old virgin male or female psyllids were released in the center of the arena. As a criterion for data collection from each insect, five minutes were allowed for a response (first choice). In the case of a response, 10 min was allowed to observe the final choice as well as the residence time in each one of the four odor fields. The assays run from 15:00 to 18:00, according to the diel rhythm of mating activity^6^.

### Experiment 1. Field evaluations of attractants as potential ACP lures

These assays were carried out in the same organic grove with natural *D*. *citri* infestation as described in experiment 1, from January 3 to March 20, 2019. For this study, yellow sticky cards (30 cm in length × 10 cm in width) with a 2 cm in diameter central hole were used to assess the attractiveness of formic, acetic, and propionic acids to *D*. *citri* adults. Each compound was tested comparing two concentrations, and naked yellow sticky cards (without attractive compound) as control (C). The following experiments were carried out: formic acid (0.025 µg × 0.25 µg × C; 0.25 µg × 2.5 µg × C; and 0.0025 µg × 25 µg × C), acetic acid (0.1 µg × 1 µg × C; 1 µg × 10 µg × C; and 0.01 µg × 100 µg × C), and propionic acid (0.01 µg × 0.1 µg × C; 0.1 µg × 1 µg × C; and 0.001 µg × 10 µg × C). Tested compounds were diluted in hexane and 100 µL were loaded into slow-release devices made of ES fiber (Ethylene-Propylene Side by Side, Chiso Co. Ltd, Japan). The assays were designed in 3 × 3 Latin square using 9 traps spaced 25 m from each other (3 traps per treatment). The assays were repeated from 12 to 24-fold over time and the number of captured ACP males and females was recorded every 24 h for 9 days.

### Experiment 2. Field tests of slow-release formulations

These assays were conducted in unsprayed ‘Valencia’ citrus orange groves located at California State Polytechnic University, Pomona, California, USA, from September 15–25, 2018. An acetic acid slow-release device (ChemTica-A) consisting of a brown polyethylene bag of 5.5 cm width x 3.5 cm length with enough load to last at least 15 days was manufactured by ChemTica Internacional S.A. (Santo Domingo, Costa Rica) and compared with our acetic acid homemade release device (1 µg). Devices were attached in a central hole (2 cm in diameter) made in the traps. Naked yellow sticky cards (without devices) were used as a negative control. These assays were carried out following a 3 × 3 Latin square design repeated 21-fold over time, totaling 63 replicates per treatment. The ACP male catches were recorded every 24 h for 10 days.

### Experiment 3. Evaluation of lures for possible synergy

These assays were carried out in the same organic grove with natural *D*. *citri* infestation as described in experiment 1, from March 25 to April 26, 2019. Lures were prepared with the following blends: formic acid (0.025, 0.25, 0.75, 1.50, or 2.25 µg) blended with acetic acid (1 µg), and propionic acid (0.01 µg) blended with acetic acid (1 µg). Lures with only acetic acid (1 µg) and naked yellow sticky cards (without attractive compound) were used as positive and negative controls, respectively. These experiments were performed with 8 replicates. The number of captured ACP males was recorded every 24 h for 9 days.

### Statistical analyses

All statistical analyses were done with Prism 8 (GrapPad, La Jolla, CA). Each pair (treatment vs. control) in indoor behavioral measurements was compared by using two-tailed, Wilcoxon matched-pairs signed-rank test (*P* < 0.05). Field tests with multiple comparisons (different doses, binary mixtures, and formulation) were analyzed by Kruskal-Wallis, followed by Dunn’s multiple comparison tests (*P* < 0.05).

## Supplementary information

Supplementary Information

## References

[1] Bove JM (2006). Huanglongbing: A destructive, newly-emerging, century-old disease of citrus. J Plant Pathol.

[2] da Graca JV (2016). Huanglongbing: An overview of a complex pathosystem ravaging the world’s citrus. J Integr Plant Biol.

[3] FUNDECITRUS. *United againts greening (in Portuguese)*, unidoscontraogreening.com.br (2017).

[4] Anonymous. *Citrus Pest & Disease Prevention Program 2017*–*2018 Annual Report*, http://www.citrusinsider.org/annual-report-2018/ (2018).

[5] George J, Robbins PS, Alessandro RT, Stelinski LL, Lapointe SL (2016). Formic and acetic acids in degradation products of plant volatiles elicit olfactory and behavioral responses from an insect vector. Chem Senses.

[6] Zanardi OZ (2018). Putative sex pheromone of the Asian citrus psyllid, Diaphorina citri, breaks down into an attractant. Sci Rep.

[7] Vet LEM, van Lenteren JC, Heymans M, Meelis E (1983). An air-flow olfactometer for measuring olfactory responses of hymenopterous parasitoids and other small insects. Physiol Entomol.

