# Supplementary material for: Laboratory and field evaluation of acetic acid-based lures for male Asian citrus psyllid, Diaphorina citri
Source: Sci Rep. 2019 Sep 9;9:12920. doi: 10.1038/s41598-019-49469-3 (PMC6733878; doi:10.1038/s41598-019-49469-3)
Supplement: Supplementary file 1 — Supplementary Information [file 41598_2019_49469_MOESM1_ESM.pdf]

Supplementary Information

Laboratory and field evaluation of acetic acid-based lures for male Asian citrus psyllid, *Diaphorina citri*

Odimar Z. Zanardi[a,1], Haroldo X. L. Volpe[a,1], Rejane A. G. Luvizotto[a], Rodrigo F. Magnani[a], Francisco Gonzalez[b], Carolina Calvo[b], Cameron A. Oehlschlager[b], Benjamin J. Lehan[c], Victoria Esperança[a], Jennifer Y. Delfino[a], Renato de Freitas[a], Rômulo Igor de Carvalho[a], Tatiana Aparecida Mulinari[a], Marcelo P. Miranda[a], José Mauricio S. Bento[d] & Walter S. Leal[e,*]

[a]Research and Development Department, Fund for Citrus Protection (Fundecitrus), Vila Melhado, Araraquara, SP, 14807-040, Brazil

[b]Chemtica International S.A, Santo Domingo, Heredia, Apdo. 640-3100, Costa Rica

[c]California State Polytechnic University-Pomona, 3801 W. Temple Avenue, Pomona, CA 91768, USA

[d]Department of Entomology and Acarology, Luiz de Queiroz College of Agriculture, University of São Paulo (ESALQ/USP), Piracicaba, SP, 13418-900, Brazil

[e]Department of Molecular and Cellular Biology, University of California-Davis, Davis, CA 95616, USA

[1]O.Z.Z. and H.X.L.V contributed equally to this work.

[*]To whom correspondence may be addressed. Email: wsleal@ucdavis.edu

Corresponding author:
Walter S. Leal
Department of Molecular and Cellular Biology
University of California-Davis
Davis CA 95616 USA
Tel: (530)-752-7755
Email: wsleal@ucdavis.edu

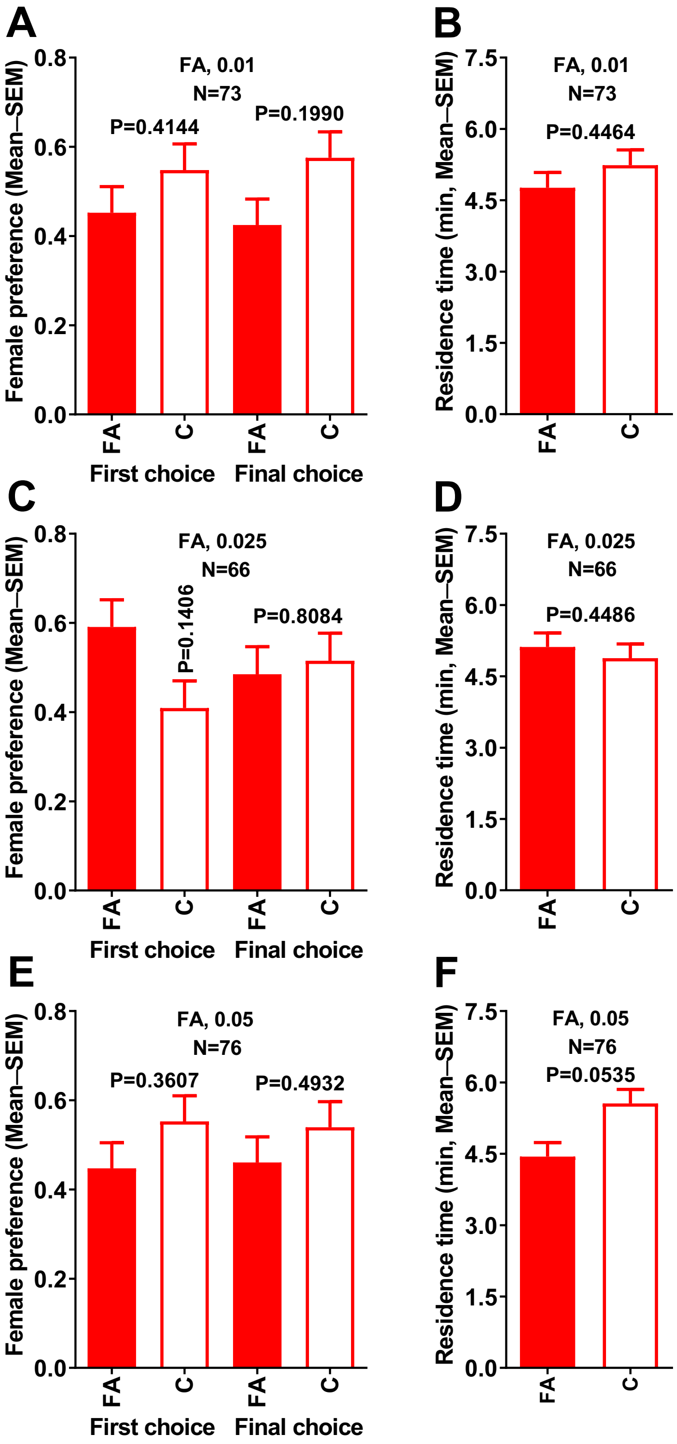

Figure S1. Behavioral responses of 7-day-old virgin females to formic acid in a 4-way olfactometer. Female first and final choices when comparing (A) 0.01, (C) 0.025, and (D) 0.05 µg of formic acid with control. Comparison of residence times in each treatment vs. control with formic acid at (B) 0.01, (D) 0.025, and (F) 0.05 µg. Means were compared by Wilcoxon matched-pairs signed rank tests. FA = formic acid.

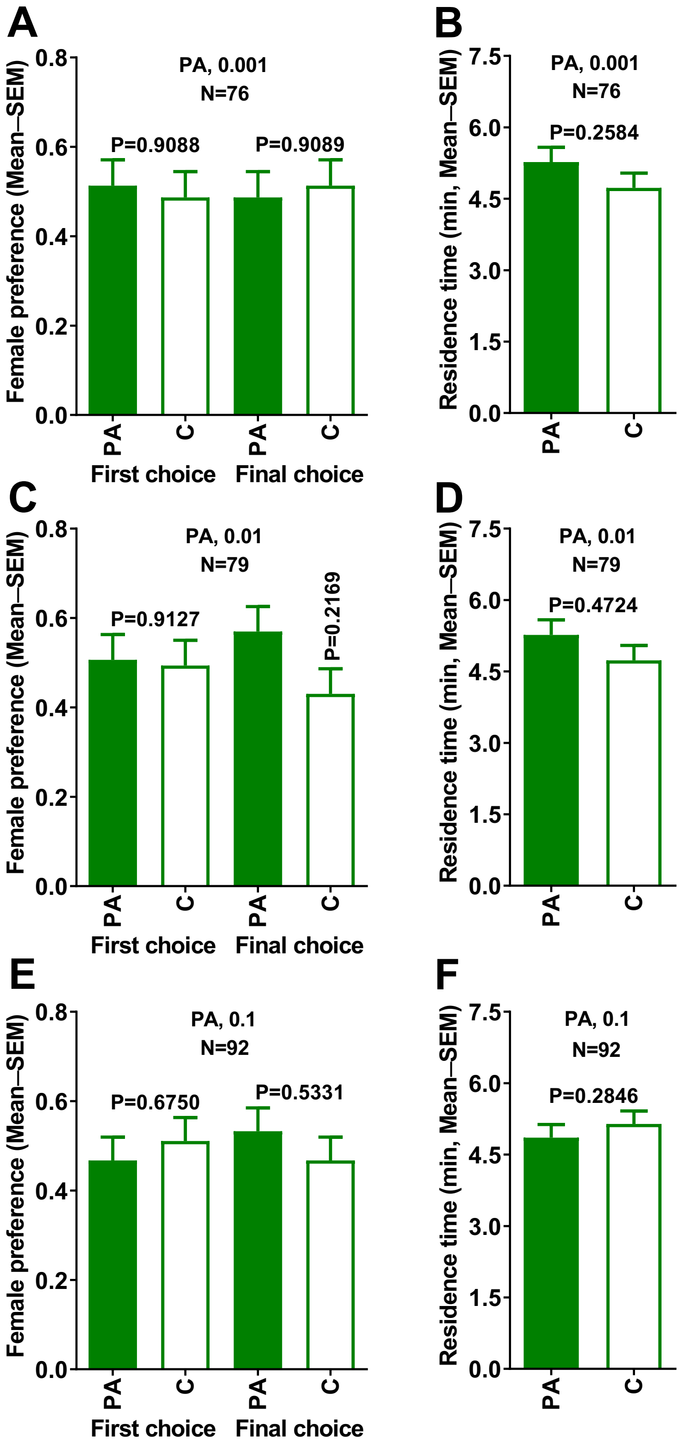

Figure S2. Behavioral responses of 7-day-old virgin females to propionic acid in a 4-way olfactometer. Female first and final choices when comparing treatments with (A) 0.001, (C) 0.01, and (D) 0.1 µg of propionic acid vs. control. Comparison of residence times in each treatment vs. control with propionic acid at (B) 0.001, (D) 0.01, and (F) 0.1 µg. Means were compared by Wilcoxon matched-pairs signed-rank tests. PA = propionic acid.

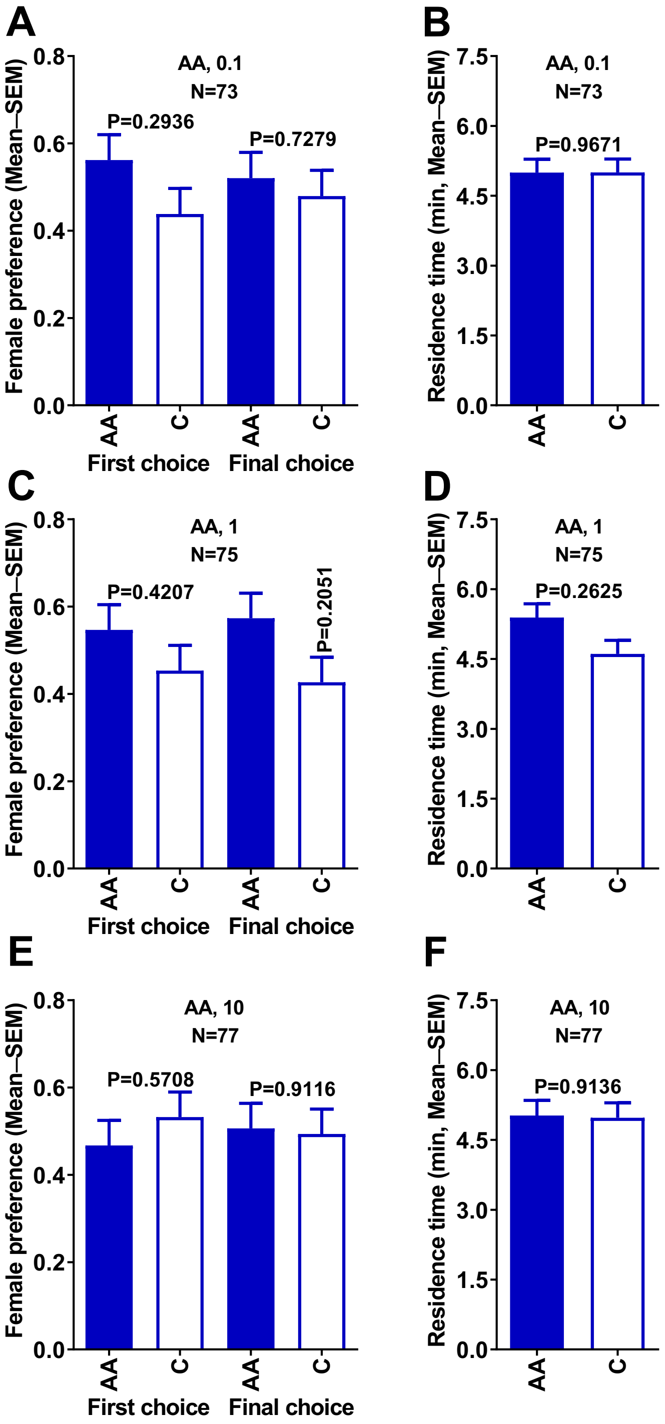

Figure S3. Behavioral responses of 7-day-old virgin females to acetic acid in a 4-way olfactometer. Female first and final choices when comparing treatments with (A) 0.1, (C) 1, and (D) 10 µg of acetic acid vs. control. Comparison of residence times in each treatment vs. control with acetic acid at (B) 0.1, (D) 1, and (F) 10 µg. Means were compared by Wilcoxon matched-pairs signed rank tests. AA = acetic acid.

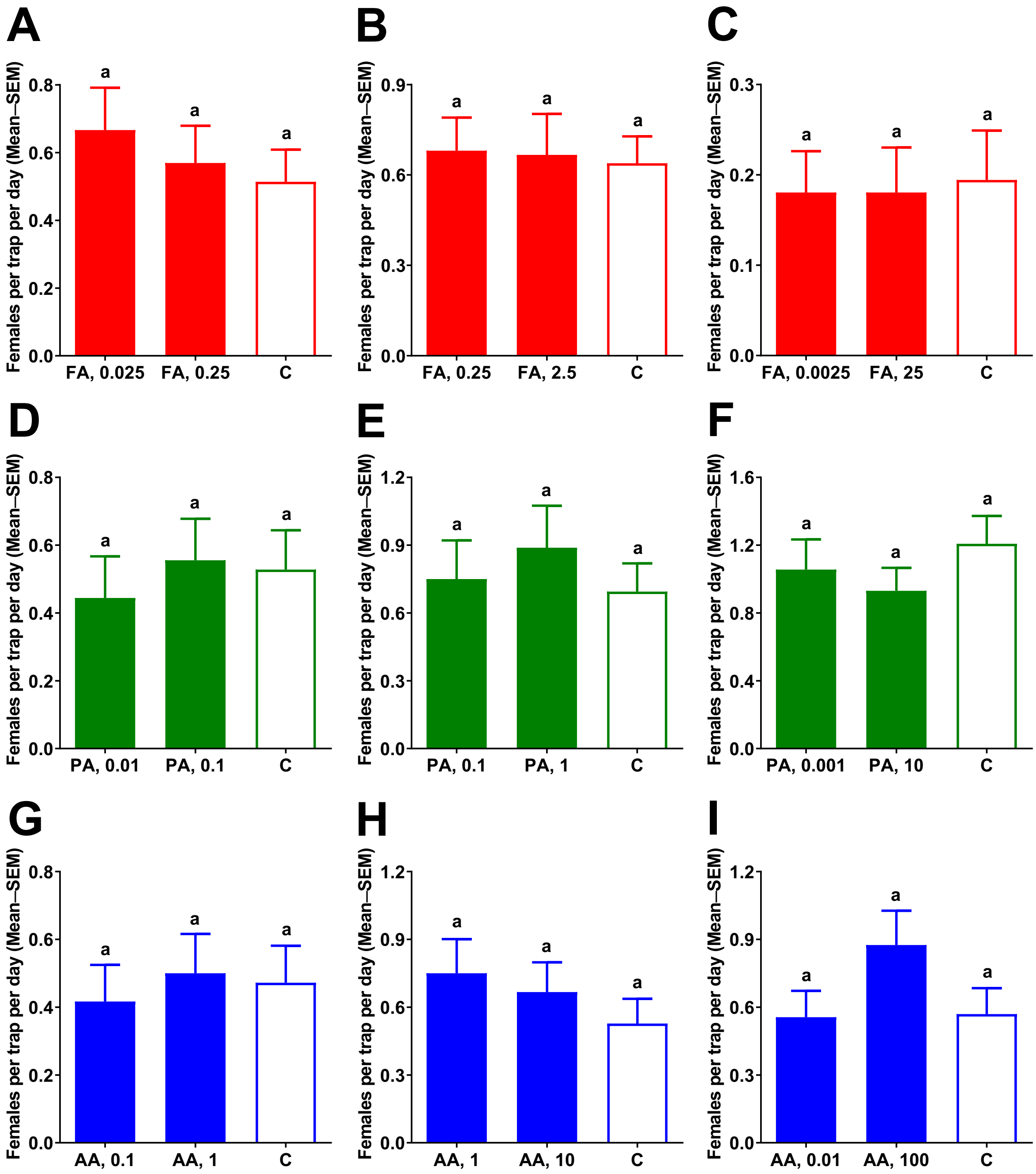

Figure S4. Field evaluations of ACP attractants as potential lures. Female captures in traps baited with formic acid (FA) at (A) 0.025 and 0.25 µg; (B) 0.25 and 2.5 µg; (C) 0.0025 and 25 µg; propionic acid (PA) at (D) 0.01 and 0.1 µg; (E) 0.1 and 1 µg; and (F) 0.001 and 10 µg; and acetic acid (AA) at (G) 0.1 and 1 µg; (H) 1 and 10 µg; and (I) 0.01 and 100 µg were compared with their respective controls. Data were analyzed by Kruskal-Wallis, followed by Dunn's multiple comparison tests. In each test, bars labeled with the same letters are not significantly different.

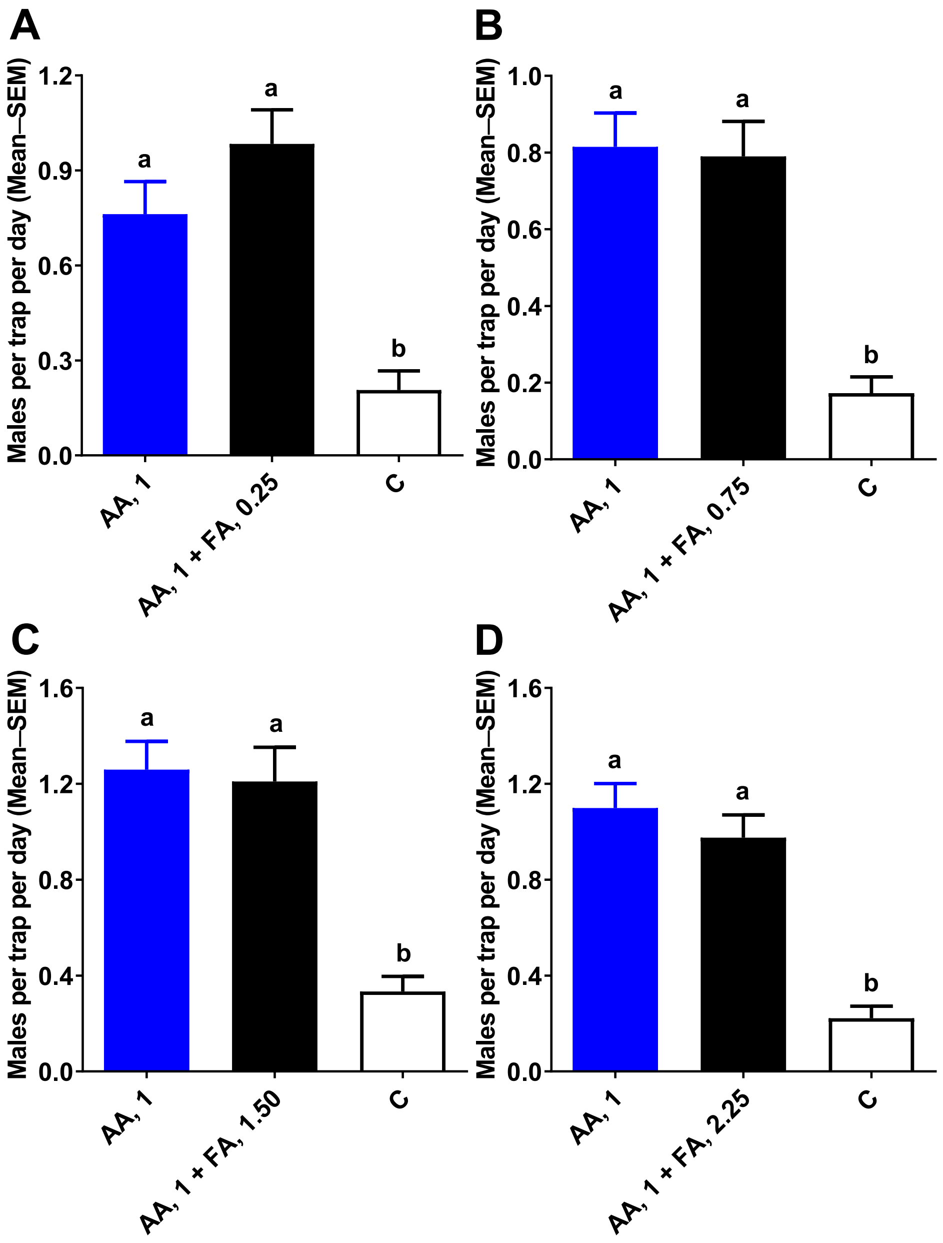

Figure S5. Field evaluations of potential synergy between acetic acid (AA) and formic acid (FA). Captures in traps baited with AA, 1 µg were compared with catches of ACP males in traps baited with (A) AA, 1 µg plus FA, 0.25 µg; (B) AA, 1 µg plus FA, 0.75 µg; (C) AA, 1 µg plus FA, 1.5 µg; and (D) AA, 1 µg plus FA, 2.25 µg; and their respective controls. Data were analyzed by Kruskal-Wallis, followed by Dunn's multiple comparison tests. For each group of experiments, bars labeled with the same letter are not significantly different.
